# Supplementary material for: Meiotic pairing through barcode-like satellite DNA repeats
Source: Nat Commun. 2026 Jun 16;17:7633. doi: 10.1038/s41467-026-74398-x (PMC13434574; doi:10.1038/s41467-026-74398-x)
Supplement: Supplementary file 2 [file 41467_2026_74398_MOESM2_ESM.pdf]

Description of Additional Supplementary Files

File name: Supplementary Data 1

Description: This file contains p values from all the comparisons made in the study.
